# A tale of three cities: uncovering human-urban interactions with geographic-context aware social media data

**DOI:** 10.1007/s44212-022-00020-2

**Published:** 2022-12-19

**Authors:** Junjun Yin, Guangqing Chi

**Affiliations:** 1 Social Science Research Institute and Population Research Institute, The Pennsylvania State University, University Park, PA 16802 USA; 2 Department of Agricultural Economics, Sociology and Education, The Pennsylvania State University, University Park, PA 16802 USA

**Keywords:** Human-urban interaction, Geographic context, Mobility pattern, Activity pattern, Geo-located tweets

## Abstract

Seeking spatiotemporal patterns about how citizens interact with the urban space is critical for understanding how cities function. Such interactions were studied in various forms focusing on patterns of people’s presence, action, and transition in the urban environment, which are defined as human-urban interactions in this paper. Using human activity datasets that utilize mobile positioning technology for tracking the locations and movements of individuals, researchers developed stochastic models to uncover preferential return behaviors and recurrent transitional activity structures in human-urban interactions. Ad-hoc heuristics and spatial clustering methods were applied to derive meaningful activity places in those studies. However, the lack of semantic meaning in the recorded locations makes it difficult to examine the details about how people interact with different activity places. In this study, we utilized geographic context-aware Twitter data to investigate the spatiotemporal patterns of people’s interactions with their activity places in different urban settings. To test consistency of our findings, we used geo-located tweets to derive the activity places in Twitter users’ location histories over three major U.S. metropolitan areas: Greater Boston Area, Chicago, and San Diego, where the geographic context of each location was inferred from its closest land use parcel. The results showed striking spatial and temporal similarities in Twitter users’ interactions with their activity places among the three cities. By using entropy-based predictability measures, this study not only confirmed the preferential return behaviors as people tend to revisit a few highly frequented places but also revealed detailed characteristics of those activity places.

## Introduction

As the world is undergoing rapid urbanization cities become the main human settlements. Citizens navigate through the landscape of urban environments for various types of daily activities, such as staying at home, traveling to work/school, and shopping at grocery stores or malls. Understanding the spatiotemporal characteristics of citizens’ interactions with the urban environments helps gain valuable insights into how cities function and reveal the city image and its elements (Lynch, 1960). The representations of such interactions vary in different forms. Based on the research themes identified in the existing literature, we propose a new concept, namely human-urban interaction, to encapsulate the interactions as people’s presence (visitation), action (activity), and transition (mobility) in urban space. Studies on human-urban interactions offer insights into the interplays between human activities and the geospatial settings of urban environments, which is of great importance to urban planning and its applications. For example, the agglomerations of citizens’ visited locations are used to identify meaningful urban places (Ahas et al., 2010); human activity patterns are used to reveal spatial–temporal urban structures (S. Jiang et al., 2012); and human mobility patterns are used to characterize the roles of street networks for predicting traffic flows (B. Jiang et al., 2009). Studies on detailed human-urban interactions rely on data sources that are capable of tracking individuals staying and moving across the urban space (Q. Huang & Wong, 2016). Owing to the proliferation of mobile positioning technology, many recent studies started to utilize GPS units (Thierry et al., 2013; Wan & Lin, 2016), mobile phones (Gonzalez et al., 2008), Wi-Fi (Sapiezynski et al., 2015), and Location Based Social Media (Grinberg et al., 2013; Shaw et al., 2016) to track the locations of individuals and use them as proxies to characterize human-urban interactions.

On the one hand, human behaviors in interacting with urban space are complex and are expected to vary in different spatial, temporal, and even cultural urban settings (Gutiérrez-Roig et al., 2016). For example, human mobility behaviors are found to play an important role in influencing different city growth patterns by shaping urban morphologies (Xu et al., 2021). On the other hand, existing studies on uncovering human mobility and activity patterns in the urban environment suggest that such behaviors can be characterized by statistical models and are common, if not universal, in different urban settings. For example, by using mobile phone location data to track people’s movements, Gonzalez et al. (2008) uncovered that human movements in urban environments show a high degree of temporal and spatial regularity, where people tend to return to a few highly frequented locations. Such patterns were also observed in intra- and inter-city travels when using geo-located Twitter data (Jurdak et al., 2015; Yin & Chi, 2021). Song et al. (2010) characterized the patterns as preferential returns and developed entropy-based predictability measures to show human movements are highly predictable. When representing people’s daily movements as mobility works, Schneider et al., (2013a, 2013b) uncovered that 17 unique networks, known as mobility motifs, are sufficient to represent up to 90% of daily urban movements in different countries. However, as most of the models are stochastic, these studies have fixated on the dimensions of the movements, which completely neglect the differences among the underlying places. Indeed, deriving the actual places that people interact with from their location history, known as activity places, remains a challenging task. Ad-hoc heuristics and spatial clustering methods were applied to derive meaningful places from the collections of locations (Pappalardo et al., 2021). However, the lack of semantic meaning in those locations makes it difficult to infer activity places beyond home and workplaces (Vanhoof et al., 2020).

In this connection, emerging studies have attempted to utilize auxiliary datasets to first infer activity places by enriching the semantics of the recorded locations of individuals. In particular, the land use types of the locations are found to be tied to different human activities, such as residential, workplace, school, leisure, and others (Widhalm et al., 2015). For example, Widhalm et al. (2015) used land use data to understand the activity types of detected places from mobile phone location data; Huang and Wong (2016) derived activity zones classified from urban land planning maps to characterize Twitter user movement flows among them; Soliman et al. (2017) integrated parcel-level detailed land use maps to understand the frequently visited places in Twitter users’ location history. Further, Yin and Chi (2021) inferred the activity types of Twitter users’ visited places and studied the transitional activity patterns. The integration of land use information essentially adds geographic context to the recorded locations, which is an important step to contextualize a person’s presence, action, and transition in the urban space.

However, the existing studies have focused primarily on the human behavior aspect of human-urban interactions. As activity places can vary drastically in different cities in terms of their spatial distributions and organizations, little is known about whether or how people’s interactions with their activity places vary in different urban settings. Therefore, this study aims to provide a better understanding of the activity places in people’s daily life by connecting the underlying spatial and temporal characteristics of human interactions. Considering the availability and effectiveness of using geo-located Twitter data for studying human activity patterns, we adopted the approach developed by Yin and Chi (2021) to obtain people’s activity places and the associated interactions in the urban environment. To ensure consistency in our findings, we conducted case studies over three major U.S. metropolitan areas from the East Coast to Midwest and the West Coast: Greater Boston, Chicago, and San Diego. In this study, we constructed the location history of Twitter users’ activities over the study areas using the geo-located tweets covering the entire year 2014, where the geographic context of each location was inferred from its closest land-use parcel. The main contribution of this study is that we examined all three aspects of human-urban interactions over three different cities. To elaborate, this study uncovered Twitter users’ visitation patterns to their activity places in the urban environment; it provided a detailed picture of the characteristics of those most frequently visited activity places; it also examined Twitter users’ preferential return behaviors in the three cities by quantitatively measuring the predictability of Twitter users’ transitions among these activity places. The results revealed striking spatial and temporal similarities in Twitter users’ interactions with their activity places across all three cities. The entropy-based predictability measures not only reaffirmed the existence of preferential return behaviors that Twitter users’ transitions among different activity places are highly predictable but also suggested that such behaviors are consistent across all three cities despite the different urban settings. This study demonstrates that geographic-context aware Twitter data can be a new and effective tool for urban planners to understand the detailed characteristics of people’s activity places in the urban environment and how people interact with their activity places, which can be particularly useful when other data sources are scarce or not even available.

## Background and related work

Cities are complex urban systems. A growing body of literature has focused on seeking spatial and temporal interaction patterns between human activities and the geospatial settings of urban environments (Bassolas et al., 2019). Such interactions are explored in various forms in the existing studies but can be generally summarized from three interconnected aspects of human activities in the urban environment: (1) visitation: citizens’ presence at physical urban locations (2) activity: actions tied to explicit activities, and (3) mobility: transitions among those urban locations or activities. Therefore, in this study, we define the term, human-urban interaction, to encapsulate the interactions as people’s presence (visitation), action (activity), and transition (mobility) in urban space. Current studies on seeking spatial and temporal human-urban interaction patterns are often conducted at the collective (e.g., focusing on the aggregated form of human activities in urban environments) or at the individual level (e.g., focusing on the activity patterns of individuals) (Barbosa et al., 2018).

At the collective level, a major research theme is to understand the agglomeration of spatial and temporal distributions of human activity locations and their connections with the functions or structures of urban areas (Jenkins et al., 2016; Liu et al., 2015; Sun et al., 2015). Multiple types of data sources were used to track people’s activity locations (i.e., presence) in the urban environment. For example, mobile phone location data were used to uncover spatial and temporal urban structures based on the density of aggregated user locations (S. Jiang et al., 2012; Niu et al., 2015). Geo-located Twitter data were used to get a collective sense of urban places by examining the alignment between the spatially clustered Twitter user locations and the physical urban places (Jenkins et al., 2016); other Location Based Social Media data such as Facebook check-ins were used to identify the urban spatial structure and urban vibrancy (T. Chen et al., 2019) and Foursquare check-ins were used to generate user movement flow to identify city centers (Sun et al., 2015). The aggregated, or spatially clustered, human activity locations were further utilized for applications in uncovering land use types and socioeconomic features in the urban environment, known as urban sensing or social sensing approaches (Calabrese et al., 2014; Liu et al., 2015). Studying human-urban interactions at the collective level offers insights into the interplays between human activities and the geospatial settings of urban environments. However, it is still difficult to dissect the agglomerations as the underlying human activities vary drastically by individuals. For example, an agglomeration can be a result of a temporary gathering for a festivity event, or it could be a commercial district, where people’s visitations to and movements from it are for different purposes.

Existing studies on human-urban interactions at the individual level have mainly focused on seeking patterns in individuals’ movements and activities in the urban environment. Conventional data sources for studying human activity patterns are collected by conducting large-scale surveys, such as travel surveys (Fairnie et al., 2016) and activity diaries (J. Chen et al., 2011). The data records often consist of information about the type, location, timing, duration, and sequencing of an activity (Kwan & Lee, 2003). For instance, travel diary data were used to (1) model individuals’ activity space (Susilo & Kitamura, 2005) (2) seek people’s daily mobility patterns (Schönfelder & Samaga, 2003), and (3) assess environmental exposures to individuals (Klepeis et al., 2001). Furthermore, the recorded activity sequence enables studies to examine transitions among different types of activities, which helps uncover the mobility motif structure in people’s daily travel networks (Schneider, Rudloff, et al., 2013). However, collecting survey-based activity data is expensive and labor-intensive to reach a large group of people or monitor their movements for a relatively long period.

Therefore, recent studies have turned to new data sources, where the locations of individuals are collected through GPS or mobile positioning technology. In particular, mobile phone location data and geo-located social media data are the two most commonly used data sources for studying human-urban interactions. The locations of mobile phone users are collected from Call Detail Records (CDR) or geolocation-enabled mobile applications. In comparison, the locations of social media users are collected when users post social media messages with the geolocation feature turned on or by tagging a message with geolocation, such as geo-located tweets and check-ins. Both data sources were used to explore population dynamics across large spatial scales. For example, mobile phone location data were used for dynamic population mapping (Deville et al., 2014) and for inferring internal migration patterns (Blumenstock, 2012). Similar studies also use geo-located Twitter data for estimating migration patterns at regional, national, and even international scales (Hübl et al., 2017; Yin et al., 2022; Zagheni et al., 2014). The ability to track the movements of a large population has enabled studies to connect human mobility with societal issues. Particularly, both data sources were utilized to assess the socioeconomic impacts of the COVID-19 pandemic through the changes in human mobility patterns (Levin et al., 2021; Chang et al., 2021; X. Huang et al., 2020).

A suite of human activity models was developed to quantitatively explain the complexity of human-urban interactions. Specifically, by using mobile phone location to track the movements of individuals, Gonzalez et al. (2008) characterized the shape of individuals’ trajectories of movements by a single spatial probability distribution, which uncovered recurrent returns in people’s movements in the urban space. The movement distances can be characterized by a truncated power-law distribution, which suggests the existence of far more short-distance movements than longer ones. Further, by treating the mobile phone users’ trajectories of movements as time series, Song et al. (2010) utilized entropy to measure the degree of predictability of movements among the visited locations, which suggested a 93% potential predictability in user movements across the user population, also known as the preferential returns behaviors. Because the high predictability of user mobility is largely independent of the movement distances, the process of human movements can be modeled by a set of Markov chain-based models (Lu et al., 2013).

### Activity location, place, and geographic context

For this study, we need to make an important distinction between two terms, namely activity location and activity place. Conceptually, the two terms are often used interchangeably. However, it will cause some confusion when dealing with different human activity datasets. For example, a unique feature in survey-collected human activity datasets is that a person’s activity (e.g., home, work, or leisure activity) is associated with an address or a named place (Axhausen et al., 2002). In those scenarios, we refer to addresses or named places as activity places. In contrast, when people’s locations are represented by 2D geographical points, we refer to those locations as activity locations. Compared to survey-collected human activity data, an intrinsic limitation exists in most, if not all, data sources collected via mobile positioning technology or GPS, that the collected locations are represented by simple 2D geographical points. The lack of semantic meaning in those locations makes it difficult to contextualize an individual’s interaction with the urban space. A variety set of approaches and methods were developed to derive meaningful activity places from the collections of locations, such as ad-hoc heuristics and spatial clustering methods, where the spatial clusters of the location history of individuals are deemed as significant places in people’s daily life (Pappalardo et al., 2021). For example, to infer home and workplaces, the spatial statistics method Getis Ord ${G}_{i}^{*}$ was used on geo-located Twitter data (Steiger et al., 2015), spatial kernel-based (Thierry et al., 2013), and visual-analytics based (Andrienko et al., 2007) methods were applied to raw GPS data. Spatial clustering methods, such as K-means (MacQueen, 1967), DBSCAN (Ester et al., 1996), and Gaussian mixture models (GMM) were used on mobile phone location data and geo-located Twitter data (S. Jiang et al., 2018; Jurdak et al., 2015; Kang et al., 2005). Despite these approaches being likely to cause algorithmic uncertainty (Kwan, 2016), the lack of semantic meaning in those locations makes it difficult to infer activity places beyond home and workplaces (Vanhoof et al., 2020).

Therefore, recent studies started to utilize auxiliary datasets to enrich the semantics of the recorded locations and differentiate the associated movements. In this line of research, human activities were found to have strong ties to the underlying land use, such as home activity (residential land use), work (commercial land use), school (educational land use), leisure (recreational land use), among others (Widhalm et al., 2015). The integration of land use information essentially enriches geographic context to the recorded locations as semantic labels. For example, Widhalm et al. (2015) used land use data to understand the activity types of detected places from mobile phone location data; Huang and Wong (2016) derived activity zones classified from urban land planning maps to characterize Twitter user movement flows among them; Soliman et al. (2017) integrated parcel-level detailed land use maps to reveal what kind of places do people tweets in Chicago, and Yin and Chi (2021) utilized parcel-level detailed land use maps to infer the activity types of Twitter users’ visited locations and studied the recurrent transitional activity patterns.

### Geo-located Twitter data for human–environment interactions

Although many studies have used mobile phone location data to study human-urban interactions, access to such data sources is still limited to the research community due to privacy concerns (Crawford & Finn, 2015; Q. Huang & Wong, 2016). Because mobile service providers or data brokers have proprietary rights to the datasets, it is difficult to conduct comparative studies across different regions. In addition, the location accuracy of mobile phone location data relies on the spatial distribution of the cell towers and can be low in the order of several kilometers (Deville et al., 2014). Many recent studies utilize publicly accessible Location Based Social Media data, such as geo-located Twitter messages (i.e., tweets) and check-in records to exploit human activity patterns, such as human travel and mobility behaviors (Hasan et al., 2013). In addition to mining social media content for social science applications, geo-located social media data are considered important sources of ambient geographical information (Stefanidis et al., 2013) and have been exploited as proxies for studying human and urban dynamics.

Among different social media platforms, Twitter offers publicly accessible data streams using Twitter Stream API (https://dev.twitter.com/api). A geo-located tweet is a regular tweet tagged with a real-world geographic location. Such a location is represented by a pair of latitude and longitude coordinates, which are usually derived from location-based service enabled smartphones via integrated GPS and Wi-Fi positioning. The geographic locations are considered to have high spatial resolution down to 10 m (Jurdak et al., 2015). Geo-located Twitter data were used to better describe the characteristics of Twitter user visited urban places (Jenkins et al., 2016). The data were proven useful for studying human mobility patterns at regional, national, and even international scales (Hawelka et al., 2014; Jurdak et al., 2015; Yin et al., 2016), and the identified mobility patterns are comparable to the ones from using mobile phone location data (Jurdak et al., 2015). The data were also used to study people’s daily activities in the urban environment by revealing the recurrent transitional activity structures (Yin & Chi, 2021). Note that there are certain limitations to using geo-located tweets as a means of tracking people’s whereabouts and exploring their activity patterns (e.g., data sampling and representativeness issues, which are discussed in the next section). However, given the spatial coverage of geo-located Twitter data, it is suitable for conducting comparative studies over different urban settings to examine detailed characteristics of actual activity places and the spatiotemporal patterns of people interacting with their activity places.

## Data and methods

### Study areas and geo-located Twitter data

In this study, we used two sets of geographic-context aware Twitter data used in Yin and Chi (2021), which were already generated for Greater Boston and Chicago. To ensure the approach for generating geographic-context aware Twitter data is valid in other regions, we selected San Diego as an additional case study area with the following two considerations. First, because Greater Boston is a metropolitan city on the East Coast of the U.S. and Chicago is in the Midwest, the third city is preferred to be a metropolitan city on the West Coast. Second, as this study intended to use parcel-level detailed land use maps to infer the geographic context of the tweet locations, the complex land use in densely built metropolitan areas can induce significant uncertainty. For example, a tall building in a downtown area can have different usages from the ground floor and up, whose land use type is often classified as “urban mix”. For this consideration, we did not choose Los Angeles or San Francisco as a study case as the mentioned situation can be severe in the two cities, whereas it is relatively less complicated in San Diego.

The data were collected using the Twitter Streaming API by setting a geographical bounding box over San Diego to retrieve all the geo-located tweets that fall in. The bounding boxes covered the three cities using the lower left and upper right coordinates for Greater Boston (41.41, -72.66; 43.12, -69.45), Chicago (41.20, -88.70; 42.49, -87.52), and San Diego (32.44, -117.49; 33.15, -116.76). Because the data collection was carried out at the city level, it did not exceed the data volume limit (i.e., 1% of the entire real-time tweet sets generated on twitter.com) mentioned in (Hawelka et al., 2014). In other words, almost all available geo-located tweets over San Diego from January 1^st^ to December 30^th^, 2014, which was the same timeframe used for the other two cities. The data collection contains over 12.5 million, 10.2 million, and 8 million geo-located tweets over Greater Boston, Chicago, and San Diego, respectively.

For each geo-located tweet, we extracted the following information: User ID, location, and timestamp, which is denoted by a tuple $\langle id,loc,t\rangle$, where $id$ is a unique string assigned to a user’s Twitter account; $loc$ is the recorded location of the tweet represented by a pair of geographical coordinates $\langle latitude,longitude\rangle$; $t$ is the timestamp of when the tweet was posted. To protect Twitter users’ privacy, the $id$ field was replaced by a randomly generated unique number. We then constructed a location history for each Twitter user (${Traj}_{id}$) by appending all the recorded locations (with the same $id$) in chronological order (sorted by timestamps), which is denoted by:

$${Traj}_{id}\equiv \left\{id;\langle lo{c}_{1},{t}_{1,}\rangle ,\langle lo{c}_{2},{t}_{2}\rangle ,\dots \langle lo{c}_{i},{t}_{i}\rangle \dots ,i=\mathrm{1,2},3\dots n\right\}$$

**Fig. 1:**
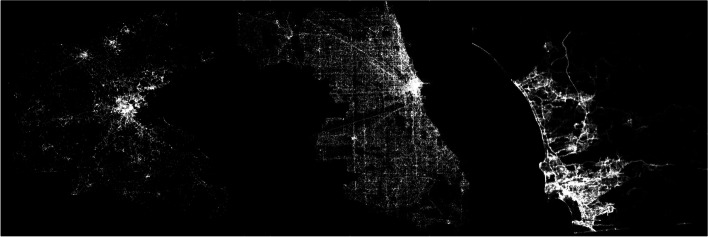
The spatial coverage of geo-located tweets over three US cities (left to right: Greater Boston Area, Chicago, and San Diego)

We applied the same perdures employed by Yin and Chi (2021) for data cleaning and preparation. Specifically, we removed non-human users based on unusual relocation speeds by examining all of the consecutive locations of each user and excluded those with relocating speeds over the threshold of 240 m/s (Jurdak et al., 2015). To reflect the activity patterns of residents rather than tourists in the three cities, we imposed a condition that the time interval between a user’s first and last recorded tweets should be more than 30 days. In other words, a user that is identified to have stayed in the study region for more than 30 days is considered a citizen. At this stage, the data contain 98,024, 87,866, and 61,238 individual Twitter users from Great Boston, Chicago, and San Diego, respectively. The spatial coverages of the filtered geo-located tweets are shown in Fig. 1, where the location of each geo-located tweet is plotted as a point. Note that the visualization reveals the geography of the three cities.

**Fig. 2:**
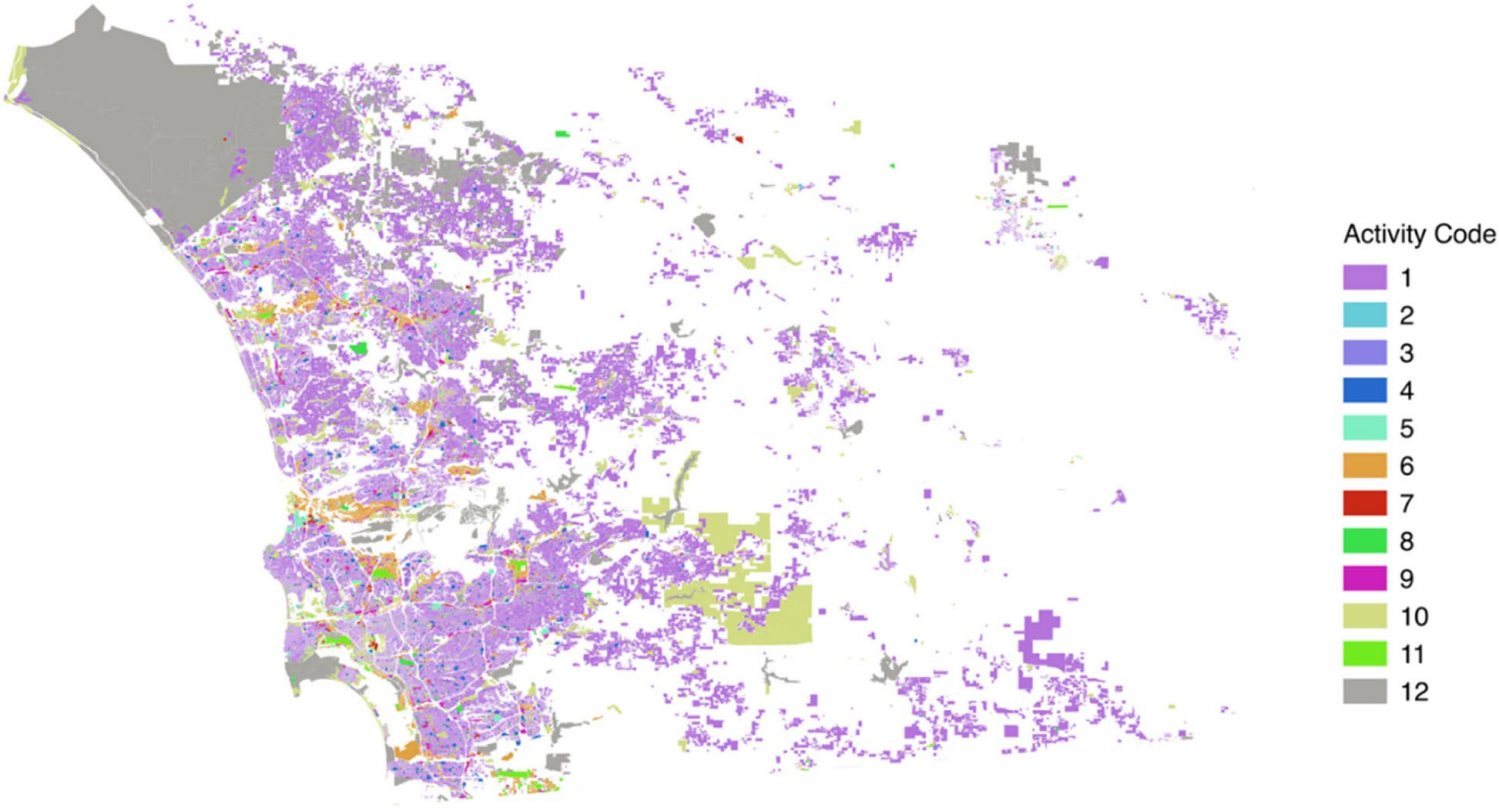
Spatial coverage of the land use parcels reclassified into 12 activity categories over San Diego

The parcel-level detailed land use maps of San Diego were downloaded from the Planning Department of San Diego (https://www.sandiego.gov/). As the geo-located tweets were collected in 2014, we extract land use parcels from the same year. The details of the parcel-level land use maps for Greater Boston and Chicago can be found in (Yin & Chi, 2021). Because land use maps are produced by local authorities, different classes and categories may be used for labeling the land use. To reflect the activities at those land use parcels, we followed the activity scheme used for Greater Boston and Chicago. Note that the “school” activity was separated into “K-12” schools and “universities/colleges” as these two activities can be vastly different from each other. The “urban mix” land use parcels, such as the residential-commercial mix, were labeled as “mixed-use”. Because the “Hotel/Resort” activities were not in travel surveys, they were listed as an individual activity class. The spatial coverage of the land use parcels of San Diego, with corresponding activity classes, are shown in Fig. 2. The relationships between the activity code and land use category and the percentage of each type of land use parcels are shown in Table 1. Note that residential land use parcels are the most prominent urban features in all three datasets.

**Table 1:** The activity code and percentage of the land use parcels in the three cities in this study

Activity Code	Land Use Category	Boston	Chicago	San Diego
1	Residential	92.65%	74.15%	78.91%
2	Hotel/Resort	0.04%	0.12%	0.48%
3	Mixed use	0.80%	12.36%	3.31%
4	K-12 Schools	0.10%	0.79%	0.67%
5	University/College	0.11%	0.15%	0.06%
6	Office/Workplace	1.36%	2.71%	3.90%
7	Services	0.56%	0.50%	0.89%
8	Civic/Religious	0.26%	1.91%	1.15%
9	Shopping/Retail	0.99%	0.07%	2.76%
10	Recreation/Entertainment	0.66%	0.85%	2.46%
11	Transportation	0.55%	3.49%	1.21%
12	Others	1.94%	2.90%	4.18%
Total number of parcels		1,117,027	164,619	108,148

### Identifying Twitter users’ activity places

In the current form, the locations in each Twitter user’s location history are still represented by 2D geographical coordinates. To derive actual activity places from each user’s location history, we first overlaid all the Twitter user locations with the parcel-level detailed land use maps. Each user location was assigned to its closest land use parcel. To account for the spatial inaccuracy of the geographical coordinates, which shifts from approximately 10 m to 250 m (Jurdak et al., 2015), we only considered candidate land use parcels that were within a 250-m search radius of the user location. The corresponding land use parcel (${p}_{i}$) and activity code (${a}_{i}$) assigned to each user location were derived from its closest land-use parcel (based on the shortest distance to the boundary of the closest land use parcel). If there were no parcels within the radius, the activity code assigned to the location is set to 12 as unknown (i.e., “other”). Every Twitter user’s location history is now transformed into an activity sequence, which is denoted as follows:

$${Traj}_{id}\equiv \left\{id;\langle lo{c}_{1},{t}_{1,}{p}_{1},{a}_{1}\rangle ,\langle lo{c}_{2},{t}_{2},{p}_{2},{a}_{2}\rangle ,\dots \langle lo{c}_{i},{t}_{i},{p}_{i},{a}_{i}\rangle \dots ,i=\mathrm{1,2},3\dots n\right\}$$

Because each location was already anchored to the nearest land use parcel, instead of using spatial clustering methods to determine spatial clusters from a user’s location history, the actual activity places are those land use parcels: (1) only those land use parcels with the tweets counts above the average number of tweets in a user’s trajectory will be considered as activity places (2) the popularity of those activity places are ranked by the number of tweets at each parcel. Essentially, this approach serves as a de-facto spatial clustering method without arbitrarily using the geometry of a spatial cluster as a place. Note that most tweeted activity places are not necessarily users’ home places. In this study, we are not fixated to identify Twitter users’ “home” places but simply focusing on understanding the characteristics of those activity places. At this stage, the land use parcel (${p}_{i}$) and activity code (${a}_{i}$) in each user’s activity sequence is updated as a corresponding activity place (${p}_{j}$) and the activity code (${a}_{j}$) of the place, which is denoted by:

$${Traj}_{id}\equiv \left\{id;\langle lo{c}_{1},{t}_{1,}{p}_{1},{a}_{1}\rangle ,\langle lo{c}_{2},{t}_{2},{p}_{2},{a}_{2}\rangle ,\dots \langle lo{c}_{i},{t}_{i},{p}_{j},{a}_{j}\rangle \dots ,i,j=\mathrm{1,2},3\dots n\right\}$$

### Predictability of Twitter users’ activity places

Given the collection of Twitter users’ activity sequences across three different urban settings, it allows us to examine, and seek patterns from, all three aspects of human-urban interactions. In particular, we aim to employ quantitative measures to examine whether the preferential return behaviors still exist among the derived activity places, and if so, how predictable are the transitions among these activity places.

First, the Twitter users’ activity sequences are time series. To incorporate the temporal features associated with the movements/transitions among different activity places, we employed the measure of first passage time introduced in (Jurdak et al., 2015), also known as return time, to measure the time interval between a user’s return to the same activity place after a period of ${\Delta t}_{fp}$. It is worth pointing out that because we used geo-located tweets to track Twitter user movements, people could post multiple messages consecutively with the same geo-location, thereby simply measuring the time interval between two activity locations can distort the time distribution. Instead, we only consider the return time between two different activity places.

To capture Twitter users’ interaction behaviors with their activity places, we examined the predictability (or randomness) of the activity sequences. To do so, we used the entropy-based measures developed by Song et al. (2010) to quantify the predictability of movements among activity places in Twitter user’s activity sequence, where entropy was used to measure the degree of predictability (or randomness) in a time series. Three types of entropy measures were introduced by Song et al. (2010): (1) random entropy ${S}^{rand}$: ${S}_{i}^{rand}={log}_{2}\mathrm{N}$, where $N$ is the number of distinct places in a user’s activity sequence. It implies the predictability of a Twitter user’s activity place if the activity places are visited with equal probability. (2) the unconditional entropy ${S}^{rand}$: ${S}_{i}^{unc}={-{\sum }_{x\in {X}_{i}}{p}_{i}\left(x\right)log}_{2}{p}_{i}\left(x\right)$, where ${p}_{i}\left(x\right)$ is the probability at which a Twitter user visited the ith place out of ${N}_{i}$ places in the activity sequence. It measures the predictability based on the frequency of each activity place within the activity sequence; and (3) the real entropy ${S}^{real}$: ${S}_{i}^{real}=-{\sum }_{{x}_{i}\in {X}_{i}}{p}_{i}\left({X}^{^{\prime}}\right){log}_{2}{p}_{i}\left({X}^{^{\prime}}\right)=-{\sum }_{{x}_{t}\in {X}_{i}}{\sum }_{{x}_{t-1}\in {X}_{i}\cap Z}{p}_{i}\left({x}_{t-1},{x}_{t}\right){log}_{2}{p}_{i}\left({x}_{t}|{x}_{t-1}\right)$, where ${p}_{i}\left({X}^{^{\prime}}\right)$ is the probability of finding a time-ordered subsequence ${X}^{^{\prime}}$ in the time series. The real entropy not only considers the occurring frequency of each activity place but also the order in which the activity places are visited, which is deeply linked to the spatiotemporal characteristics of Twitter users’ presence and transitions among their activity places. Intuitively, ${S}_{i}^{real}\le {S}_{i}^{unc}\le {S}_{i}^{rand}$. Song et al. (2010) utilized Fano’s inequality to calculate the predictability $\prod$ associated with each entropy. The maximum bound of a user’s predictability ${\prod }_{i}\le -{\prod }_{i}^{max}\left(S,{N}_{i}\right)$, where ${\prod }_{i}^{max}$ is given by $S=H\left({\prod }_{i}^{max}\right)+\left(1-{\prod }_{i}^{max}\right){log}_{2}\left({N}_{i}-1\right)$, and the binary entropy function *H* is given by: $H\left({\prod }_{i}^{max}\right)=-{\prod }_{i}^{max}{log}_{2}\left({\prod }_{i}^{max}\right)-\left(1-{\prod }_{i}^{max}\right){log}_{2}$). Considering the assumption made for calculating random entropy, it is unlikely to be the case for practical human activities in the urban environment. Therefore, we only considered the unconditional entropy and the real entropy in this study.

## Results

**Fig. 3:**
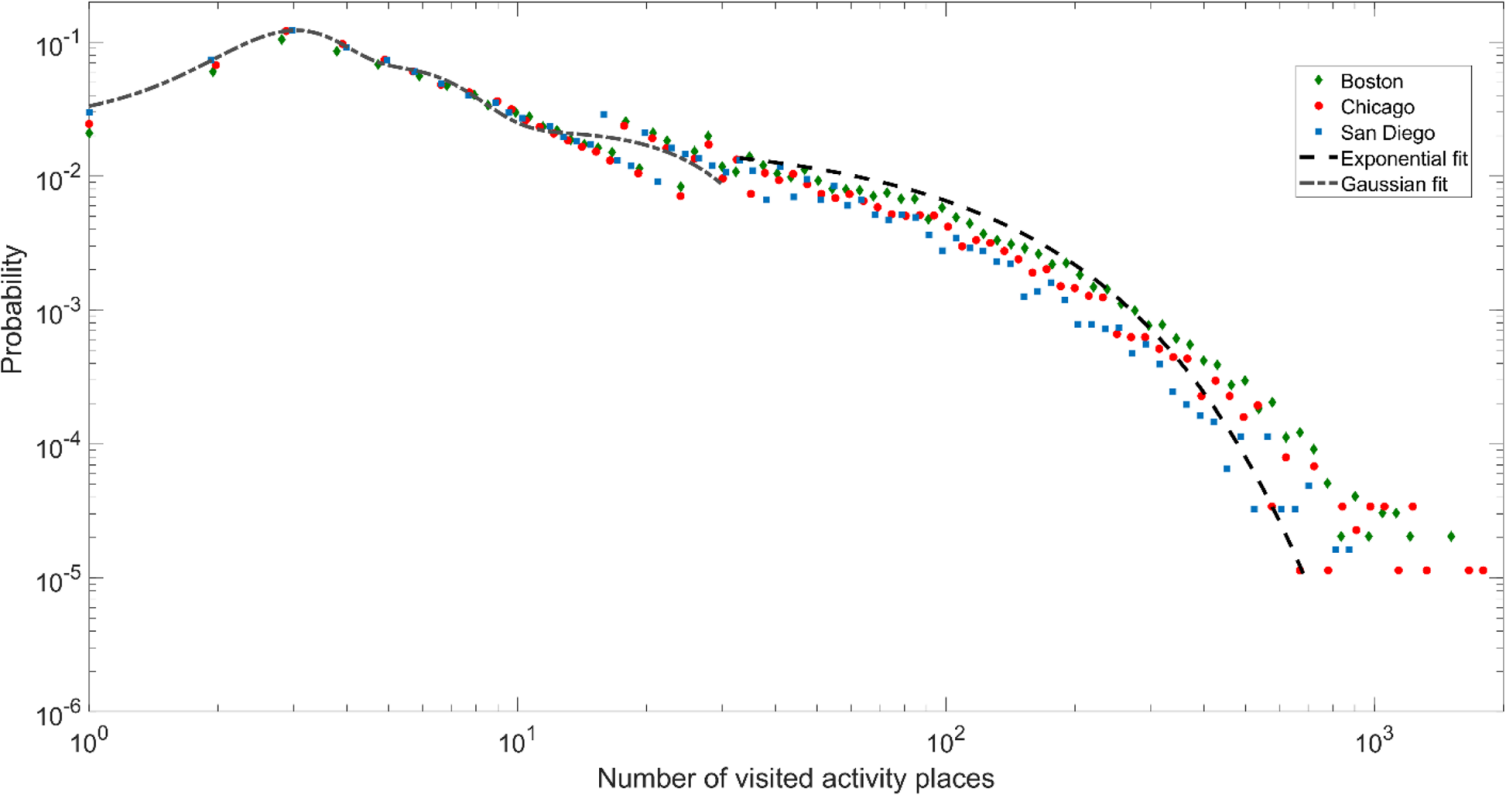
Probability density function of the number of activity places visited by Twitter users

Every citizen is a unique individual. Intuitively, the activity places people interacted with on a daily basis or over time should vary drastically by individuals as well. It may be the case when we took a first glance at the number of activity places in each Twitter user’s activity sequence. Yet, when plotting the probability density function of the number of visited activity places over the three cities in 2014, there is a clear bimodal distribution that divides the user base into two distinct groups (illustrated in Fig. 3). Specifically, for the first group of Twitter users with less than 20 activity places in their activity sequences, the numbers of visited activity places tend to follow a Gaussian distribution with a peak value of 3. Note that the visited activity places are the land use parcels at which Twitter users posted more than the average of all the land use parcels in 2014. It does not mean Twitter users only tweet at those activity places. However, the situation is far more dispersed for the second group of Twitter users with more than 20 activity places, where the numbers of visited activity places tend to follow an exponential distribution. Interestingly, the probability density functions are very similar over the three cities. It suggests that there are drastic variations involved in individuals’ behaviors regarding how many activity places people visit, but there are also strong similarities collectively (i.e., city-wide) when viewing the overall trend as a whole.

**Fig. 4:**
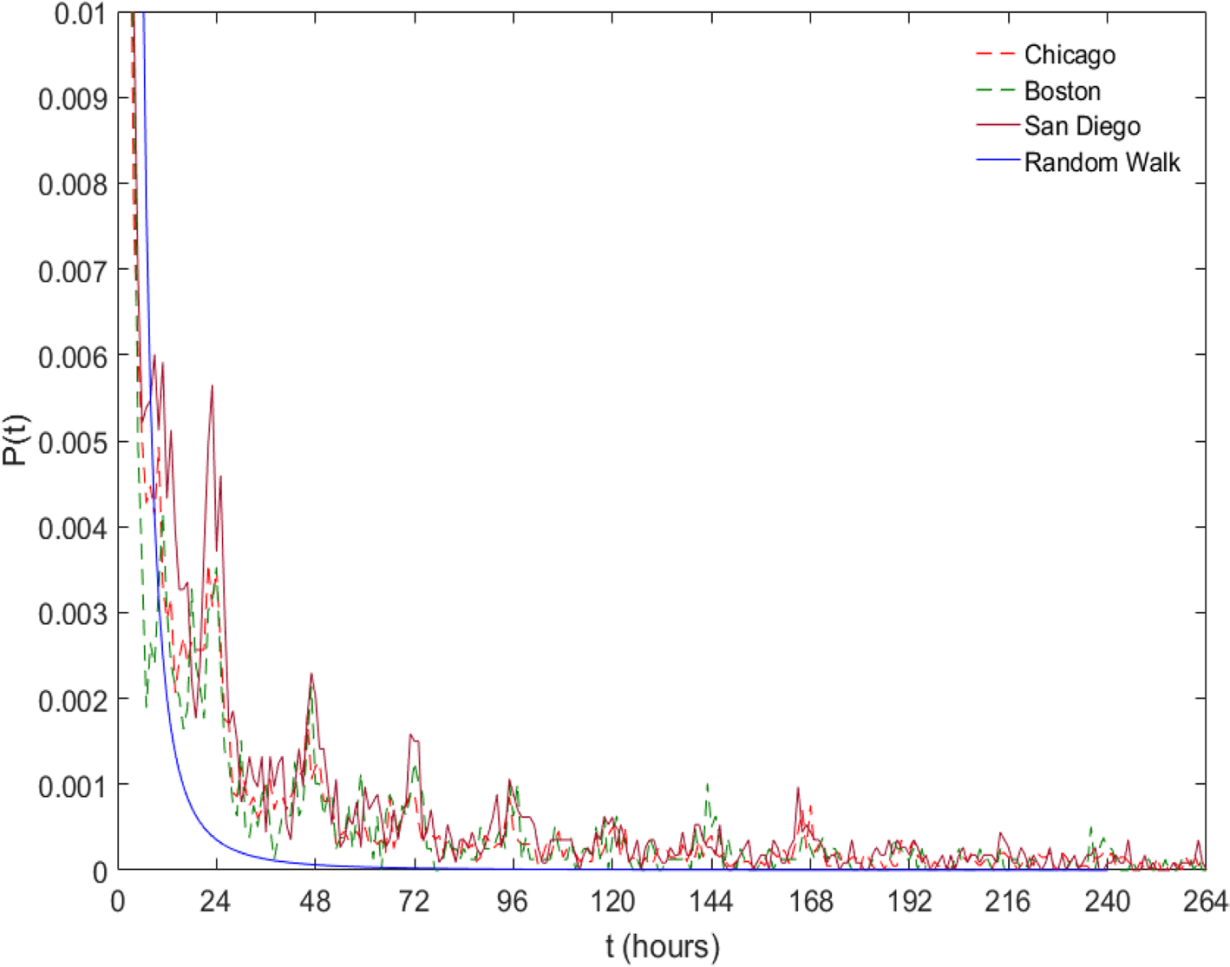
The probability distribution of the return time. Note that there are apparent periodical return behaviors in Twitter users’ return to the same activity place

To explore the temporal characteristics of Twitter users’ transitions among their activity places, we plotted the probability distributions of the first passage time in Fig. 4, which measures the probability of finding a Twitter user at the same activity place after a period of t. To assess whether the return time was the result of a random process, we added a time series generated from a random walk model as a reference. Figure 4 shows the probability distribution of Twitter users’ return time to the same activity place across the three cities. The probability distributions show that Twitter users’ behaviors in returning to the same activity place are not a random process but exhibit an apparent periodical shift with approximately a 24-h interval. In addition, the first passage time patterns are remarkably similar across the three cities. This not only reaffirms the existence of preferential return behaviors that were observed in the mentioned studies using mobile phone location data but also suggests that such behaviors are prevalent despite different urban settings.

**Fig. 5:**
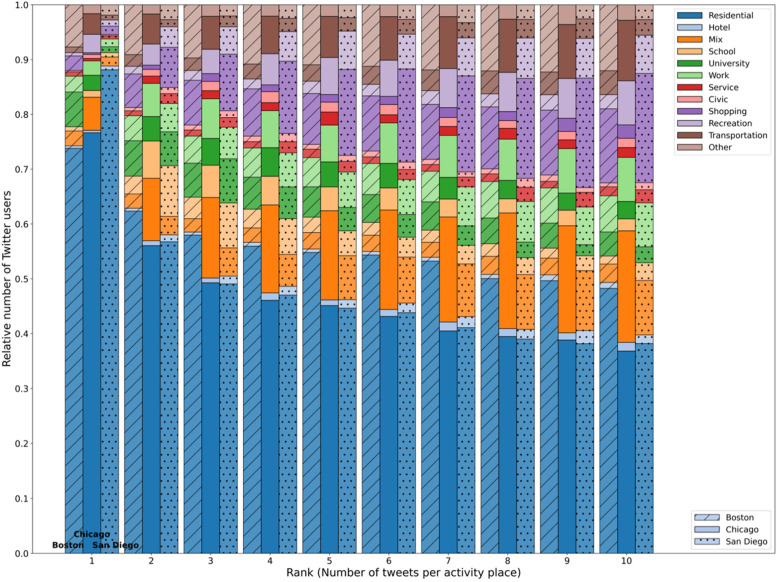
Decomposition of the top 10 most frequently visited activity places in the three cities

There are two major benefits of using land use parcels to enrich the geographic context of the locations in geo-located tweets. First, the activity places are land use parcels. Second, each activity place is assigned an activity code. Therefore, we can examine the detailed characteristics of those activity places. Specifically, we can decompose both the land use and activity types of the frequently visited activity places. Figure 5 illustrates the decomposition of land use categories in the top 10 most frequently visited activity places (ranked by the number of tweets per activity place) across the three cities. The results show that majority of all the most frequently visited activity places are residential, which is not surprising considering the land use maps show that residential land use parcels are the most prominent urban features in all three cities. Particularly, in the most frequently visited (or equivalently, the most tweeted) activity places across the three cities, over 75% of them are residential places (over 85% in the case of San Diego). This provides some evidence to support the assumption of using spatial clustering methods for detecting users’ activity places, where the most significant spatial cluster is assumed to be a user’s “home” place. Note that these residential places are not necessarily a Twitter user’s “home” place (e.g., it could be a vacation home). Nevertheless, we can observe that some Twitter users do tweet the most at non-residential places, such as universities and workplaces, which suggests the user base in this study does cover a diverse Twitter user population. As the rank decreases, the percentages of other non-residential activity places increase indicating that Twitter users do tweet regularly or substantially at a diverse collection of urban places.

**Fig. 6:**
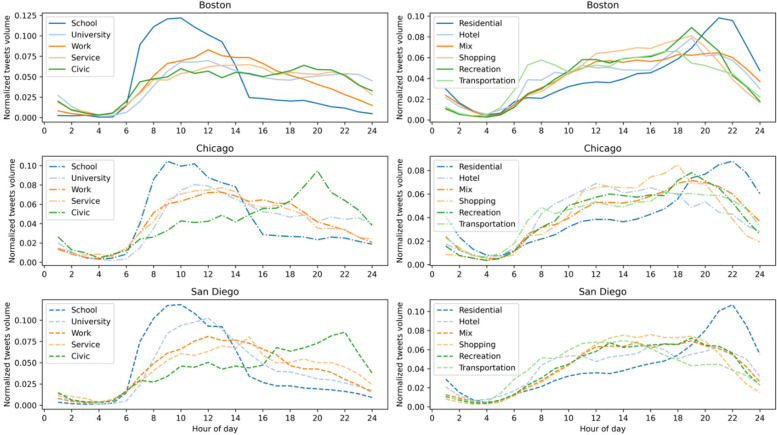
Weekday hourly distribution of tweets volumes at Twitter users’ activity locations classified by different land use types

It is worth pointing out that the activity sequences not only capture the whereabouts of Twitter users, which is tied to 1 out of 12 activities, but also the timing of the whereabouts. Therefore, we utilized the intensity of Twitter users’ weekday activities (measured by tweets volume) at different times of the day to get a better sense of the temporal characteristics of people’s activities in the urban environment. The weekday hourly distribution of tweets volumes at Twitter users’ activity places classified by different land use types is shown in Fig. 6. The tweet volumes were normalized within each land use category. The intensity of Twitter users’ activities fluctuates throughout the day and varies among different types of activity places and appears to be tightly connected to the function of the activity places. For example, activities, such as at work, university, and K-12 school, reach the peak before mid-day, and gradually decrease afterward (except for K-12 school activity, which drops sharply after 3:00 PM as most students are off school then). In contrast, several activities are observed to reach the peak in the evening, such as activities related to civic, urban mix, recreation, and shopping, which are mostly observed between 6:00 PM and 8:00 PM, whereas the activities at residential places reach the peak at the latest at 10:00 PM. Notice that activities related to transportation have two peaks with a smaller one around 8:00 AM and a larger one around 6:00 PM (it is less apparent in San Diego), which may suggest its correspondence to rush hours in the cities.

The results provide insights into the connections between human activities and the geospatial setting of the urban environment. Importantly, a remarkable resemblance of the temporal characteristics of the activity places is observed across all three cities. However, there are some apparent discrepancies in the temporal signals derived from using geo-located Twitter data. First, the inference of Twitter users’ whereabouts is strongly influenced by varying human behaviors. For example, there are significantly fewer activities observed in the early morning. It does not mean people are not staying at home (or other places), but simply are not posting tweets. In the case of work activities peaking around mid-day, it seems that the signals from tweet volumes are delayed because people are less likely to tweet during early working hours but during breaks (e.g., lunch breaks).

The calculation of real entropy ${S}^{real}$ requires continuous observation of people’s activity places (e.g., hourly, or daily), as it not only considers the occurring frequency of each activity place but also the order in which the activity places were visited in a given period. However, the whereabouts of a Twitter user can only be observed when this user posts, which is similar to how mobile phone location data are collected. The varying tweeting behaviors will lead to a significant number of incomplete movements/transitions among these activity places not to be considered in the calculation. Therefore, we relaxed the time requirement and only considered the order in which an activity place was visited. The real entropy was estimated by using a Lempel–Ziv algorithm for searching repeated sequences as suggested by Jurdak et al. (2015).

**Fig. 7:**
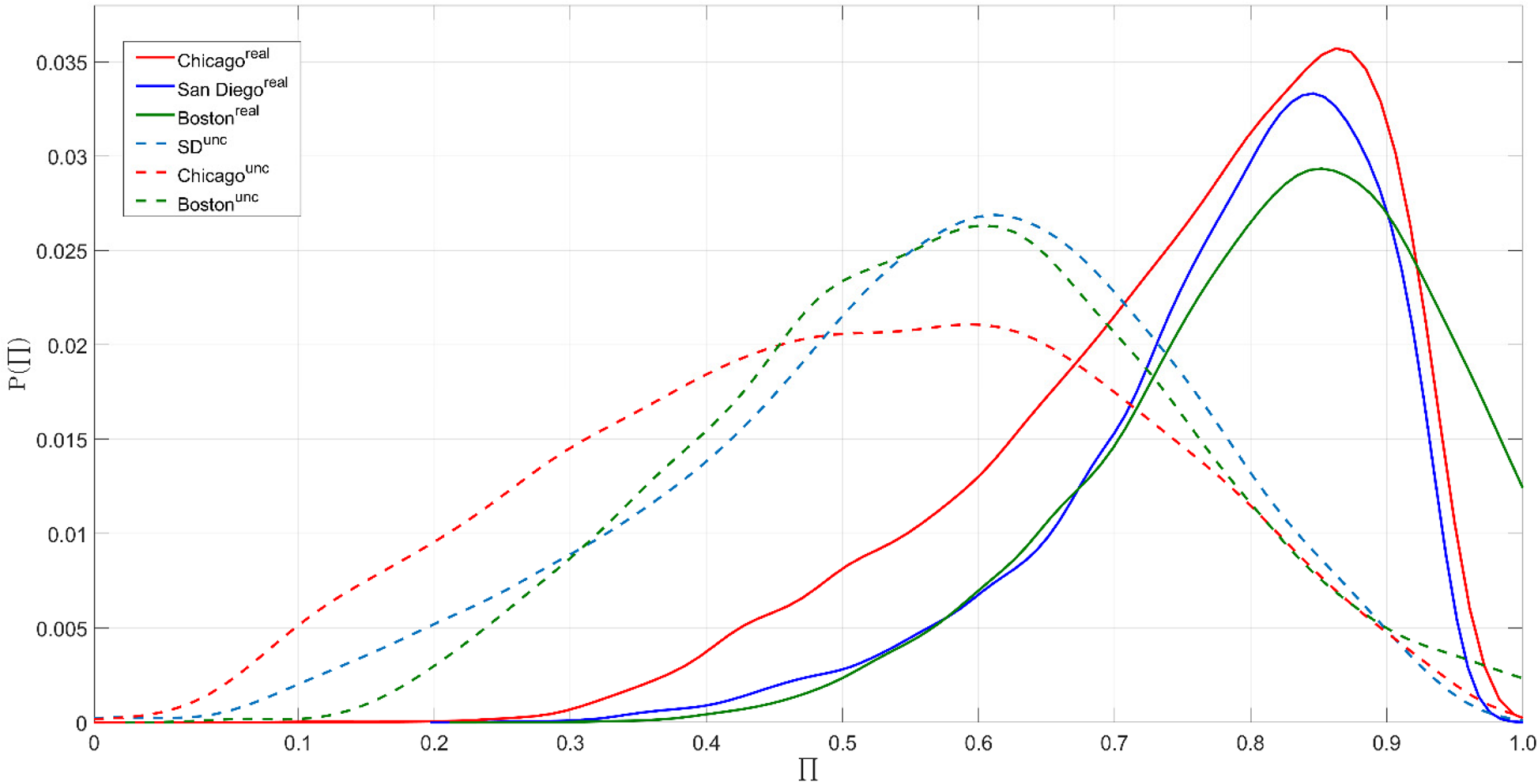
The probability density functions of the predictability of Twitter users’ activity places

The probability density functions $P(\prod )$ of the predictability of Twitter users’ activity places are shown in Fig. 7, where the dash lines represent the predictabilities ${\prod }^{unc}$ estimated from the unconditional entropy ${S}^{rand}$ and the solid lines represent the predictabilities ${\prod }^{real}$ estimated from the real entropy ${S}^{real}$. The results show that both $P({ \prod }^{unc})$ and $P({ \prod }^{real})$ reach the peak at a center predictability value. In all three cities, $P({ \prod }^{unc})$ has a broader shape than $P({ \prod }^{real})$ with a majority of the predictability values around a center predictability value at 0.6. It suggests that, if only considering the occurring frequency of activity places in a Twitter user’s activity sequence, the potential predictability for the next activity place these users would visit is 0.6 (the highest predictability is 1). In comparison, $P({ \prod }^{real})$ has a tighter shape with a majority of the predictability values around a center predictability value at 0.85, which highlights the importance of the additional consideration of the order of visitation sequences in predicting the next activity places. The probability density functions $P(\prod )$ are consistent across the three case study cities, even though the Twitter users’ activity sequences were generated from different user bases and different urban settings.

## Discussions and conclusions

It is the rhythm in everyday citizens’ daily activities that forms the heartbeat of the city. The ability to capture and understand how citizens interact with the urban space can provide important insights to reveal how cities function. With the increasing availability of various forms of human activity data, such as surveys, mobile phone location data, and geo-located social media data, many studies have been conducted to seek different types of human activity patterns in the urban environment. However, specific to citizens’ interactions with the urban space, most of the existing studies have focused on seeking patterns either from the human aspect or from the urban space aspect of the interactions. In this connection, we proposed a new concept, namely human-urban interaction, which takes the literal meaning of the term “interaction” to encapsulate the interactions as people’s presence (visitation), action (activity), and transition (mobility) in urban space. Using this concept, this study aims to gain insights into the interplays between human activities and the geospatial settings of urban environments.

Abundant research efforts have utilized mobile phone location data and geo-located social media data for tracking the locations and movements of individuals to study human activities in the urban environment. However, the actual places that people interact with were often overlooked, where the activity places were inferred by applying ad-hoc heuristics and spatial clustering methods. The inability to identify different types of activity places from single-layered location data is mainly attributed to the absence of geographic context in the recorded locations. In this study, we utilized geographic context-aware Twitter data to investigate the spatiotemporal characteristics of people’s interactions with their activity places in different urban settings. Specifically, we used geo-located tweets and parcel-level detailed land use maps to derive activity places from their location history and constructed activity sequences over three major U.S. metropolitan areas from the East Coast to Midwest and the West Coast: Greater Boston, Chicago, and San Diego.

The results showed remarkable spatial and temporal similarities and consistencies in Twitter users’ interactions with their activity places across three different cities. First, the identified bimodal distribution in the number of activity places in Twitter users’ activity sequences divided the user base into two distinct groups. Second, Twitter users’ behaviors in returning to the same activity place exhibited a clear periodical shift with approximately a 24-h interval, which supported the existence of preferential return behaviors in human-urban interactions. Further, we examined the detailed characteristics of Twitter users’ activity places. By decomposing the top 10 most frequently visited activity places in the three cities, we found that Twitter users do tweet regularly or substantially at a diverse collection of urban places. Each type of activity place seems to show an almost unique signal to reflect the corresponding human activity in the urban environment. Using the temporal signals as indicators to understand the urban environment is very much in alinement with the concept of social sensing (Liu et al., 2015). The predictability of Twitter users’ activity sequence calculated by real entropy suggests that Twitter users’ transitions among different activity places are highly predictable. For the majority of the Twitter user base in this study, the potential predictability for predicting the next activity place they would visit is as high as 0.85 (the highest predictability value is 1).

A simple takeaway message for the tale of three cities about human-urban interactions is that although citizens' activities in the urban environment vary drastically by the individual, however, the way they interact with the urban space may be more similar/common than people think. Yet, this study provides several significant implications for urban scientists and planners for understanding how citizens interact with their surrounding urban environments. First, this study demonstrates that geographic-context aware Twitter data can be a new and effective data source for understanding the detailed characteristics of people’s activity places in the urban environment and how people interact with and transition among these activity places, which can be particularly useful when other data sources are scarce or not even available. Second, considering that people tend to return to previously visited activity places, urban planners can better plan future transportation options by evaluating the visitation patterns to existing urban infrastructures. Finally, as the transitions among different activity places are highly predictable despite different urban settings, urban scientists can consider it for smart city applications, such as for developing a universal simulation model for better resource allocations or for developing effective crisis response plans.

However, this study also shows some limitations in using geo-located Twitter data as proxies to track people’s whereabouts. Specifically, the inference of Twitter users’ whereabouts is strongly influenced by the variations in people’s behaviors when using mobile phones or Twitter. For example, people may not use the Twitter app during certain times of the day, which will result in delayed signals (e.g., in workplaces) or missing signals (e.g., in the early morning). More importantly, neither mobile phone data nor geo-located Twitter data can track user locations continuously, which means the intermediate user locations between two tweets may not be recorded. This will significantly affect our ability to derive actual or timely movements from users’ location history, which is demonstrated by calculating the real entropy value of Twitter users’ activity sequences. Although the Twitter user base in this study covers different groups of people in the urban environment, studies have shown that there is a mismatch between the Twitter user population and the real population. Particularly, the Twitter user population is skewed toward younger users (Greenwood et al., 2016). Therefore, the identified human-urban interaction patterns from this study are likely not fully applicable to the whole city population.

## Data Availability

The data and source code that support the findings of this study are available upon request. The geo-located Twitter data are not publicly available according to the Twitter data sharing policy as precise geo-locations could compromise the privacy of Twitter users. However, the activity sequences from anonymized Twitter users in the three case study cities can be made available.
